# *TNFAIP3* gene rs7749323 polymorphism is associated with late onset myasthenia gravis

**DOI:** 10.1097/MD.0000000000006798

**Published:** 2017-05-19

**Authors:** Hong-Wei Yang, Yanchen Xie, Yuan Zhao, Liang Sun, Xiaoquan Zhu, Shuhui Wang, Yong-Qiang Zhang, Ping Lei, Yunxiao Meng

**Affiliations:** a Geriatric Ward of Neurology, Tianjin Geriatrics Institute, Tianjin Medical University General Hospital, Tianjin; b Department of Neurology, Beijing Friendship Hospital, Capital Medical University; c The Key Laboratory of Geriatrics, Beijing Hospital & Beijing Institute of Geriatrics, Ministry of Health; d Department of Pathology, Peking Union Medical College Hospital, Chinese Academy of Medical Science, Beijing, China.

**Keywords:** late onset, myasthenia gravis, single nucleotide polymorphisms, susceptibility, tumor necrosis factor α-induced protein 3

## Abstract

In this study, we intended to genotype 2 single nucleotide polymorphisms (SNPs) of tumor necrosis factor α-induced protein 3 (*TNFAIP3*) genes and explore an association of *TNFAIP3* genetic polymorphism with the patients of myasthenia gravis (MG) at clinical level. In brief, 215 of adult MG patients were divided into subgroups according to their clinical features, age of onset, thymic pathology, and autoantibodies. Two hundred thirty-five of healthy controls were also divided into subgroups with gender- and age-matched. The allele and genotype frequencies of subgrouped patients were found to be higher than those of healthy controls. The distribution of *TNFAIP3* gene rs7749323∗A allele of late onset MG (LOMG, with positive acetylcholine receptor antibody and without thymoma) subgrouped patients was also significantly higher than that of gender- and age-matched healthy controls (7.4% vs 2.4%, odds ratio [OR] = 3.27, 95% confidence interval [CI] 1.01–10.6, *P* = .04). Furthermore, analysis to the genotype frequencies indicates that the carriers of rs7749323∗A allele of LOMG group became more frequent than that of age-matched healthy controls (14.9% vs 4.8%, OR = 3.47, 95% CI 1.04–11.6, dominant model: *P* = .03). It is interesting to notice that there is no significant association between the rs7749323 and susceptibility of other MG subgroups. Therefore, it is suggested that the SNPs in the 3′ flanking region (rs7749323) of *TNFAIP3* gene and the genetic variations of *TNFAIP3* gene may take an important role in the susceptibility of LOMG.

## 1 Introduction

Myasthenia gravis (MG) is an autoimmune disorder, which is principally caused by the pathogenic autoantibodies.^[1]^ The pathogenic autoantibodies are also regarded as important factors to direct toward the skeletal muscle acetylcholine receptor (AChR) at the neuromuscular junction. In general, MG is characterized by postsynaptic blockade of nervous transmission, which may result in a weakness and easy fatigue of striated muscle. As a result, the MG patients can be divided into subgroups according to their clinical features, age of onset, and thymic pathology, or according to their autoantibodies.^[2]^

Although the precise origin of autoimmune response in the MG patients so far is not completely clear, it is believed that the genetic predisposition may influence the patients who have such a disorder.^[3–6]^ Tumor necrosis factor α-induced protein 3 (*TNFAIP*3, also known as A20), which is a potent anti-inflammatory signaling molecule, can restrict and terminate the inflammatory responses through the modulation of ubiquination status of central components for the nuclear factor κB (NF-κB), interferon regulatory factor 3, and apoptosis signaling cascades.^[7,8]^

From the human genetic studies, it is known that the polymorphisms in the *TNFAIP3* gene may associate with the susceptibility of multiple autoimmune diseases,^[9]^ including systemic lupus erythematosus (SLE),^[10,11]^ rheumatoid arthritis (RA),^[12,13]^ psoriasis,^[14]^ Crohn disease,^[15]^ as well as other autoimmune diseases.^[9,15]^ But to our knowledge, there is no report on the association of *TNFAIP3* genetic polymorphisms with the MG disease. We hypothesized that the generic variants in the *TNFAIP3* gene may have an association with the MG, and in the current report, we performed a research to explore the association of polymorphisms in the *TNFAIP3* gene with MG, and furthermore examine the relationship between the generic variations of *TNFAIP3* gene and clinical manifestations for this disease.

## 2 Subject and methods

### 2.1 Study population

This is a case–control study. From July 2005 to July 2008, 215 adult MG patients were enrolled from the Tianjin Medical University General Hospital and Beijing Friendship Hospital, Capital Medical University of China and furthermore performed with median follow-up of 28 months. For the sample size, we have used the maximal samples as we could get during the study.

The healthy controls were enrolled consisting of 235 healthy individuals (111 males and 124 females) during the same period in the 2 hospitals with gender- and aged-matched to the MG population. All patients and healthy controls were northern Han Chinese and nonconsanguineous. The study was approved by ethical committees of 2 hospitals with the approval number of BJFH/2012-02-09 (Board: Hospital Ethics Committee of Tianjin Medical University General Hospital, Medical Ethics Committee of Beijing Friendship Hospital, Capital Medical University). Oral informed consent was obtained from all participants. Individual identities were described in ways that authors who had access to information would not be able to identify the participants during and after data collection.

According to criteria in the early publication,^[16]^ the MG patients were diagnosed on the basis of their clinical history, evidence of fatigue on the physical examination, exclusion of alternative diagnoses as well as a positive result at least 1 of 3 criteria: increased serum level of anti-AChR antibody (Ab), decremental response to low-frequency repetitive nerve stimulation, or positive response to neostigmine test. During the median follow-up process of the patients, based on the criteria, 158 (73.49%) of patients were identified as generalized myasthenia gravis (GMG) muscle involvement, 55 (25.58%) of patients as ocular myasthenia gravis (OMG) muscle involvement, and 2 (0.9%) of patients were found to be lack of available data. The MG patients with the thymoma were confirmed by the pathological test or imaging technique. The sets of ratio for the healthy controls are approximately equal.

### 2.2 Blood sample collection

The whole blood samples from the MG subjects and healthy controls were collected and injected into the anticoagulant treated tubes containing ethylene diamine tetra acetic acid. The blood cells were collected at the bottom of tube with a refrigerate centrifugation at 1500×g for 10 min. The platelets were removed from the plasma with a centrifugation at 2000×g for 15 min. Both the blood cells and plasma samples were stored at −80°C for the final evaluation uses.

### 2.3 Antibody testing

The antibody test against AChR in the plasma was performed using ELISA kit (RSR Limited, Cardiff, UK) and the protocol followed the instruction on the kit.^[17]^ The blood samples of 211 patients from total 215 of patients were run for the AChR Ab test. The binding capability of plasma antibody with the AChR was interpreted with the inhibition rate as listed in Table 1.

**Table 1:**
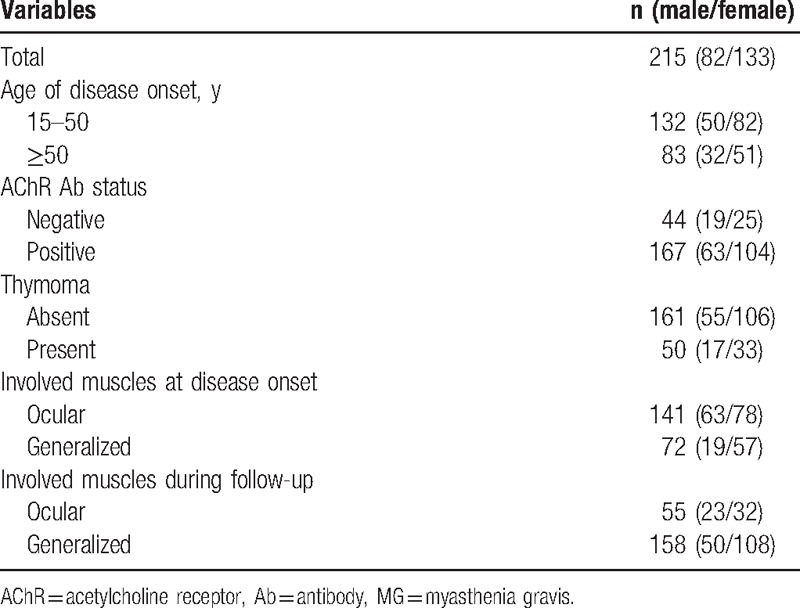
Clinical characteristics of 215 patients with MG.

### 2.4 SNP selection and genotyping

According to previous publication on genome-wide association studies (GWAS), 2 types of single nucleotide polymorphisms (SNPs) (rs5029939 and rs7749323) are believed to have positive associations with the immune-mediated disease.^[10]^ In this case, both the rs5029939 and rs7749323 were selected to perform the experiments and the results were listed in Table 2.^[8,10]^

**Table 2:**
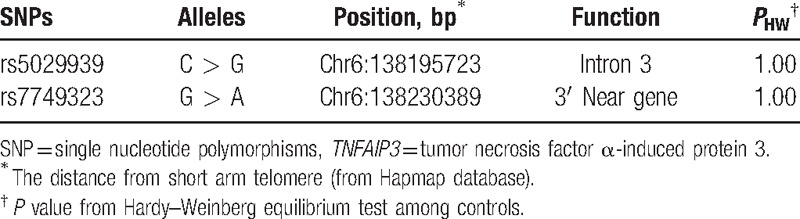
General characteristic of SNPs in *TNFAIP3* genes.

Briefly, following the instruction from the vendor (TIANGEN Biotech LTD, Beijing, China), the DNA samples were extracted from the peripheral white blood cells of the patients and healthy controls. The SNP of rs5029939 was genotyped on a polymerase chain reaction (PCR)-based restriction fragment length polymorphism analysis. The primers for amplifying SNP rs5029939 had sequences of forward primer 5′ GCC TTC ACC AGC AAA TCA AG 3′ and reverse primer 5′ GAC ACC AAC TGC AAA GGA GCC AG 3′. The PCR product (186 bp) was digested with *Afl*II enzyme and 2 fragments were yielded with 142 and 44 bps, respectively, for SNP rs5029939 CC genotype. On the 4% agarose gel, only one band of 186 bps for the GG genotype was observed supporting our viewpoint.

The SNP of rs7749323 was genotyped using allele-specific PCR technique. Two pairs of primer were designed that corresponded to the alleles. The G allele corresponded to a 120 bp length product of F1 (5′ CTA AGA CAT CAC CTC TGA TCG TGG ATG CG 3′) and R1 (5′ CTC AGT TCC TGT CTT TAT CCA GCC TC 3′), and the A allele corresponded to F2 (5′ CTA AGA CAT CAC CTC TGA TCG TGG ATG CA 3′) and R2 (5′ CTC AGT TCC TGT CTT TAT CCA GCC TC 3′). The product from F1/R1 was GG genotype, and the product from F2/R1 was AA genotype. If the product was yielded from both F1/R1 and F2/R1, the individual was defined as GA genotype.

To assurance the assay quality in our experiments, 25 of blood samples were randomly selected for the genotyping performance by Sanger-based sequencing. The concordance rate for the duplicate genotyping was found to be 100% for all our assay tests representing that the results obtained from our assays were reliable.

### 2.5 Statistical analysis

Statistical analysis was performed with SPSS Statistics 13 (SPSS Corporation, Chicago, IL). Continuous variables were presented as mean ± standard deviation and comparisons in 2 groups were performed using Student *t* test. Categorical variables, the Hardy–Weinberg equilibrium of the polymorphisms, and genotype/allele frequencies were presented with the number (percentage) and tested with the chi-squared or Fisher exact test. SHEsis software (http://analysis2.bio-x.cn/SHEsisMain.htm) was used to compare the allele frequencies and haplotype frequencies. The Haploview 4.2 software was used to calculate pairwise linkage disequilibrium (LD) of SNPs and construct haplotype blocks. The sample size and statistical power was determined using the Genetic Power Calculator (http://pngu.mgh.harvard.edu/purcell/gpc/cc2.html). The data from the case report forms were compiled as a dataset loading into a database and validated prior to the statistical analysis. The database, constructed using SQL server, contains the data pertaining to the SNPs, clinical features and treatment, and clinical follow-up of patients.

## 3 Results

### 3.1 Demographic and clinical data of participants

In this study, there were 215 MG patients (61.86% female) enrolled, and their biological and clinical features were listed in Table 1. The mean age of disease onset for these patients was 45.28 ± 16.42 years. Among these patients, 4 (1.86%) patients were found to be lack of their AChR antibody (Ab) data, so they were removed from the final evaluation. As a result, 211 patients’ AChR Ab data were available, including 167/211 (77.7%) patients were seropositive (63% in OMG and 84.5% in GMG, respectively). In addition, 161/211 (76.30%) were diagnosed as GMG and 50/211 (23.70%) were diagnosed to have thymoma on the basis of clinical criteria.

**Figure 1:**
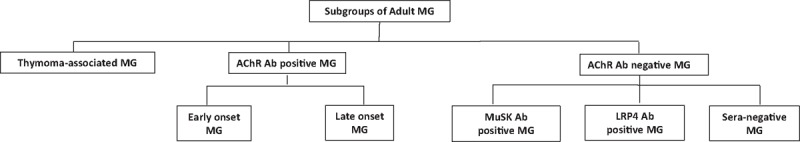
Subgroups of adult myasthenia gravis. Ab = antibody, AChR = acetylcholine receptor, LRP4 = low-density lipoprotein receptor-related protein 4, MG = myasthenia gravis, MuSK = muscle-specific tyrosine kinase.

These MG subjects were divided into the subgroups according to their clinical features, age of onset, thymic pathology, and autoantibodies. To avoid allocating 1 patient into multiple subgroups and facilitate comparison among the patients from different subgroups, a subgrouping scheme was designed as shown in Fig. 1. The subgroup division may promise the allocation of MG patients into fewer subgroups and with higher consistency.^[18]^ Typically, 211 of MG patients were divided into 4 subgroups. Fifty (23.25%) patients were identified as thymoma-associated MG (TAMG), 71 (33.02%) as early onset MG (EOMG), 49 (22.79%) as late onset MG (LOMG), and 41 (19.07%) as AChR Ab negative MG.

The ethnically matched healthy controls included 235 subjects (52.77% female) with a mean age of 49.57 ± 18.40 years.

### 3.2 Genotype and allele frequency distribution

Among 215 MG subjects and 235 healthy controls, the success rate for the genotyping of 2 SNPs was found to be more than 98.44%. Genotypes of the 2 SNPs in the healthy controls were consistent with Hardy–Weinberg equilibrium (*P* = 1.0 for both SNPs, Table 2).

The distributions of 2 SNPs in the MG subjects and healthy controls are listed in Table 3, showing that there is no significant statistical difference in the rs5029939 and rs7749323 frequency of allele and genotype between the MG subjects and healthy controls (Table 3).

**Table 3:**
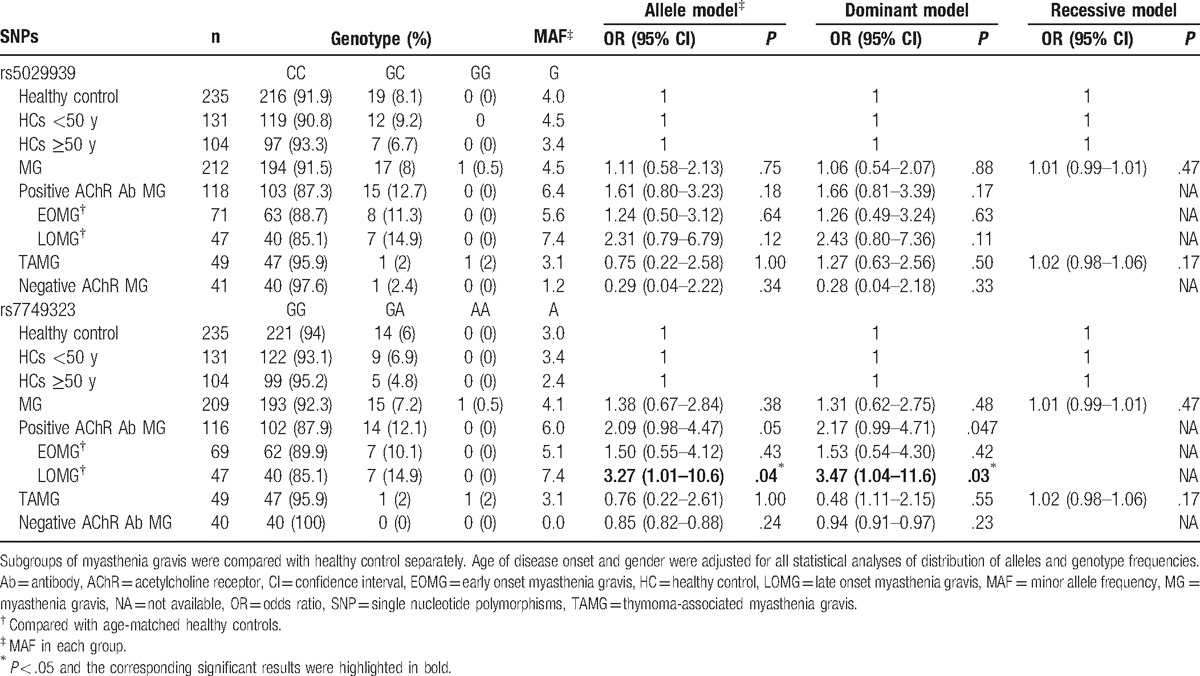
Genotypes and minor allele frequencies in patients with myasthenia gravis and the control group (%).

### 3.3 Association of *TNFAIP3* gene rs5029939 and rs7749323 with the MG subgroups

To study the precise effect of SNPs in the different population, the MG patients were divided into 3 subgroups: TAMG, AChR Ab positive MG (without thymoma), and AChR Ab negative MG (without thymoma) (Fig. 1).^[18]^ The positive AChR Ab MG subjects were further subdivided into EOMG and LOMG according to 50 years of age as the cut-off for disease onset.^[19]^ The subgroups of MG subjects were compared with those of healthy controls. The age of disease onset and gender were adjusted for all statistical analyses when considering the distribution of alleles and genotype frequencies.

It is noticed from Table 3 that the distribution of *TNFAIP3* gene rs7749323∗A allele was significantly higher in the LOMG group in comparison with that of age-matched healthy control group (age ≥ 50 years old) (7.4% vs 2.4%, odds ratio [OR] = 3.27, 95% confidence interval [CI] 1.01–10.6, *P* = .04). Further analysis to the genotype frequencies indicates a close association of the rs7749323 with the LOMG group. Carriers of rs7749323∗A allele are found to be more frequent in the LOMG group than that in the age-matched healthy control group (14.9% vs 4.8%, OR = 3.47, 95% CI 1.04–11.6, dominant model: *P* = .03). The statistical power was 53.33% for rs7749323 considering type I error 0.05. It is also interesting to notice that there is no significant association of rs5029939 SNPs with the susceptibility of any subgroups.

### 3.4 Association analysis for *TNFAIP3* haplotypes

**Figure 2:**
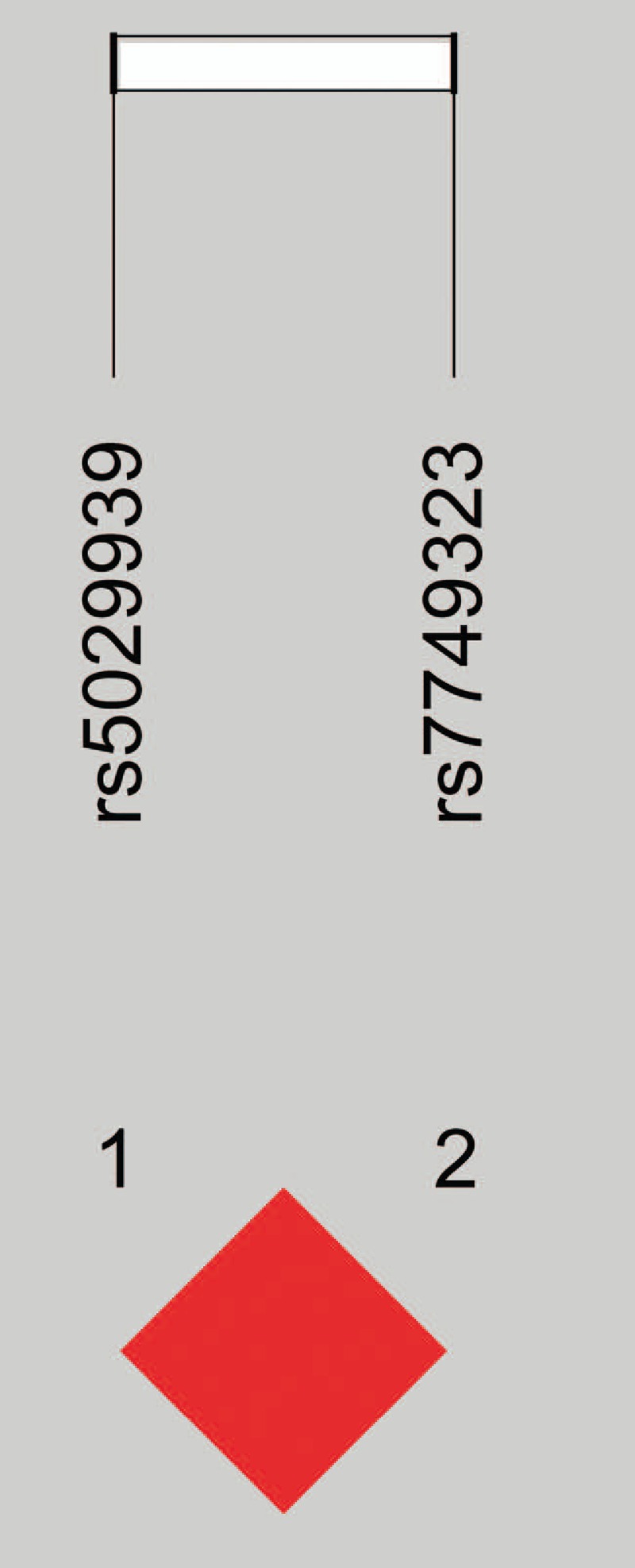
*TNFAIP3* gene haplotypes. Rs5029939 and rs7749323 linked closely (D′ = 1, *r*^2^ = 0.81).

To investigate the association of *TNFAIP3* haplotypes with the MG subjects, the LD test between the 2 SNPs in the *TNFAIP3* gene was performed. The results were listed in Fig. 2 indicating that the Rs5029939 and rs7749323 were linked closely each other (D′ = 1, *r*^2^ = 0.81) resulting in a haplotype block. Moreover, 2 common haplotypes could be identified across the block, and the frequency was estimated to be 96.0% for CG and 3.8% for GA, respectively (Table 4). One risk haplotype (GA) was identified in the LOMG group (OR = 2.59, 95% CI = 1.02–4.61, *P* = .039) which could be comparable with that in the healthy control (7.4% vs 3%). Thus, it is suggested that the risk haplotype GA and LOMG susceptibility might contain a risk allele (rs9324921∗A).

**Table 4:**
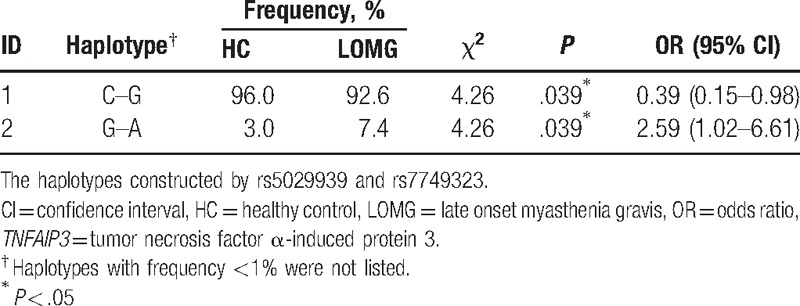
Haplotype analysis of the *TNFAIP3* gene variants between LOMG patients and healthy controls.

## 4 Discussion

In this study, the rs7749323 (locates in 3′ flanking region) of *TNFAIP3* gene is found to have an associate with the LOMG (with positive AChR Ab and without thymoma) in the northern Han Chinese population. On the other hand, there is still lack of evidence supporting the presence of an association of the MG subjects with the *TNFAIP3* gene polymorphism.

It is known that in the most MG cases, the disease is mediated by the autoantibodies targeting the muscle nicotinic AChR. In fact, the autoantibodies against AChR in an MG subject might impair a neuromuscular transmission and furthermore bring up a muscle weakness.^[2]^ According to our present study, the AChR Abs were found to presence within 84.5% of the GMG subjects, 63% of the OMG subjects, and 94% of the MG subjects with thymoma, which are close to the results in the early reports.^[20,21]^

In this study, the rs7749323 is found to closely associate with the LOMG subjects that have positive AChR Ab expression. On the other hand, although the rs5029939 is reported to associate with SLE in the GWAS,^[10]^ there is no significant difference for the allele or genotype frequency of rs5029939 in the *TNFAIP3* susceptibility genes between the positive AChR Ab group and healthy control. It is hence suggested that this genetic polymorphism might be generally restricted within the cases of some diseases or specific subgroups of a disease.

From the GWAS study, it is believed that several genetic factors may contribute to the susceptibility of MG.^[4,22,23]^ This viewpoint may be supported by the observations on the cytotoxic T-lymphocyte antigen 4 (*CTLA-4*) and human lymphocyte antigen (*HLA*)*-DQA1* polymorphism which are regarded as independent risk factors in the susceptibility of MG disease. Because of heterogeneity in the MG perplexes genetic analysis, the furthermore evidence on the susceptibility of gene for the specific subgroup of MG is still needed to support this viewpoint. So far, there are reports supporting the strong associations of LOMG with the tumor necrosis factor Receptor Superfamily Member 11a (TNFRSF11A) rs4574025 (OR = 1.42 [1.24–1.63], *P* = 3.91 × 10^−7^), zinc figer- and BTB domin containing 10 (ZBTB10) rs6998967 (OR = 0.53 [0.42–0.65], *P* = 8.86 × 10^−10^), and HLA region rs111945767 (OR = 0.52 [0.44–0.61], *P* = 3.13 × 10^−17^) in the European population,^[4]^ as well as with the protein tyrosine phosphatase nonreceptor 22 gene (*PTPN22*) R62W (OR = 3.1 [1.2–8.2], *P* = .03)^[6]^ and the HLA region rs111256513 (OR = 2.22 [1.2–8.2], *P* = 2.48 × 10^−6^) in the Turkey population.^[24]^ There are also reports on a significant association of EOMG with the HLA region rs113519545 (OR = 5.71 [3.77–8.88], *P* = 2.24 × 10^−16^) in the Turkey population and the *CTLA-4* gene in the Han population of northern China.^[24,25]^ Our finding in the present study reveals a new association of *TNFAIP3* gene with the LOMG in the northern Han Chinese population which is never reported.

The *TNFAIP3* gene can encode the protein A20, which involves in the pathogenesis of autoimmune diseases.^[7]^ Several publications have appeared to identify the *TNFAIP3* gene as a susceptibility locus for the human autoimmune pathology.^[8,9,15,26–28]^ The *TNFAIP3* gene is also regarded to closely associate with the autoimmune diseases. However, for the 2 SNPs in the present study, such an association of *TNFAIP3* gene with the MG subjects was observed only in the LOMG. Contrarily, there is no significant difference on the cumulative numbers of risk alleles and genotype frequencies for the other subgroups of MG. Particularly, there is no significant evidence to support an association of rs7749323 with the TAMG, although the AChR Abs were found to express in 94% of TAMG subjects. The results indicate that the rs7749323 gene may play an important role only in the susceptibility of LOMG.

It should be worthy to notice that only 2 SNPs in *TNFAIP3* genes were selected to perform the association study with the susceptibility of MG in this case. In addition, the present study was performed with underpowered model, which might lead to a false-negative result. Thus, to better evaluate the results from the present study and clarify the substantial role of *TNFAIP3* gene polymorphism with the susceptibility of MG, a further study is suggested to perform with a larger number of cohort and more candidates of SNP.

Currently, the MG is treated principally aiming to improve the muscle weakness, achieve the disease remission, minimize the drug-induced side effect, and slow or prevent the progression of MG.^[29]^ It includes uses of acetylcholinesterase inhibitors and immune modulating agents of corticosteroids as well as immunosuppressive therapy. A20 is known as a potent inhibitor of NF-κB signaling pathway that may take an effort in the treatment.^[7,8]^ On the other hand, although patients’ cells show increased expression of NF-κB-mediated proinflammatory cytokines, the *TNFAIP3* mutant truncated proteins are likely to act by haplosufficiency since they do not exert a dominant-negative effect in overexpression experiment.^[30]^ The current observations in our study may offer an opportunity to well understand the mechanisms of MG development and furthermore take proper medical actions to the MG treatments with different clinical features.

In summary, this study demonstrates that the rs7749323 in the *TNFAIP3* gene is strongly associated with the LOMG (with positive AChR Ab but without thymoma). Considering the association of *TNFAIP3* SNPs with the RA and SLE as well as other autoimmune diseases, it is believed that the genetic variations of the *TNFAIP3* gene may play an important role in the susceptibility of LOMG. It is also suggested to perform a furthermore study with a large number of cohort as well as more candidates of SNP to elucidate the effect of variation in the genes of *TNFAIP3* pathway on the susceptibility of MG.
